# Surveying uveitis specialists—a call for consensus

**DOI:** 10.1007/s12348-012-0061-2

**Published:** 2012-03-28

**Authors:** Emmett T. Cunningham

**Affiliations:** 1 California Pacific Medical Center, San Francisco, CA USA; 2 Department of Ophthalmology, Stanford University School of Medicine, Stanford, CA USA; 3 West Coast Retina Medical Group, 185 Berry Street, Lobby 2, Suite 130, San Francisco, CA 94107 USA

In the early 1990s, former Michigan supreme court Justice Thomas Brennan became disillusioned with popular law school rankings and so decided to survey 100 academics, judges, and lawyers on his own, asking them to rank a list of ten schools he provided. He used a composite index similar in structure, but different in content, to those used by mainstream surveyors, such as *U.S. News & World Report*. As expected, many of the big name schools—Harvard, Yale, Stanford—made it to the top of the list. Penn State, as Brennan recalled, “[Was] about in the middle of the pack. Maybe fifth among the 10 schools listed.” There was one small problem, however. Penn State had no law school at the time. Brennan had included it to make a point: surveys are limited by both the quality of the questions asked and by how familiar respondents are with the subject being surveyed [1, 2].

Similar surveys using questionnaires are employed commonly in medicine and, when well-designed and implemented, can provide valuable information regarding perceptions, preferences, and practice patterns—a finger on the pulse, if you will, of current treatment approaches [3, 4]. Almost without exception, variation is the rule in such surveys. Engstrom and colleagues, for example, published in 1991 the results of a survey of 62 treating uveitis specialists in the American Uveitis Society (AUS) regarding what were then current practices in the management of ocular toxoplasmosis [5]. While a sizable proportion of respondents viewed poor vision (<20/200), marked or severe vitreous inflammation, and zone 1 lesion location [6] as absolute indications for antimicrobial therapy, the extent to which experts reached agreement regarding when to initiate treatment was by no means absolute. Moreover, the lack of consensus was even more striking when considering which agent(s) to choose. Specifically, experts reported use of ten different antimicrobial drugs in more than four different combinations, with approximately one third recommending a combination containing pyrimethamine, folinic acid, sulfadiazine, and prednisone. Holland and Lewis updated this survey in 2002, publishing the results gathered from 79 respondents who evaluated and managed patients with ocular toxoplasmosis [7]. Similar “absolute treatment indicators” were identified, but this time 11 different agents were used in an equal number of combinations, including some not mentioned in the 1991 survey. Once again, the most common approach involved the combined use of pyrimethamine, folinic acid, sulfadiazine, and prednisone—although more than two thirds recommended other single or combination agent treatment regimens. Similar trends and variations in treatment approach were observed when uveitis specialists were surveyed in 2011 by Wakefield and associates [8]—fully 20 years after the original ocular toxoplasmosis practice survey was published. Even greater variation regarding indicators of treatment exists among non-uveitis specialists [9].

One thing well-designed surveys of medical practitioners can do particularly well is identify limitations and deficiencies in the level and understanding of current clinical knowledge, including familiarity with and adherence to published guidelines. This was nicely demonstrated by Nguyen and colleagues in 2010, when the authors published the results of a survey regarding treatment of noninfectious uveitis as reported by 60 ophthalmologists and three rheumatologists [10]. Striking among the reported findings was the extent to which treatment patterns differed from recommended guidelines published by an expert panel roughly 10 years prior [11]. This was particularly so regarding the long-term use of oral corticosteroids, which tended to be used at doses three to four times the recommended maximal dose for chronic therapy of 10 mg/day. Similarly striking was the use of corticosteroid sparing agents, which were introduced to lower systemic corticosteroid doses to or below 10 mg/day in only 12% of patients. In fact, 75% of surveyed physicians did not use and were not aware of the existence of uveitis treatment guidelines. Among the ophthalmologists surveyed by Nguyen and associates, only 19 (32%) were uveitis specialists, however, and so these results undoubtedly reflected the biases and practice patterns of the particular population surveyed.

In this issue of the *Journal of Ophthalmic Inflammation and Infection*, Esterberg and Acharya provide additional insights into the use of corticosteroid-sparing immunomodulatory therapy for chronic noninfectious uveitis [12]. The authors summarize responses from a group of 45 uveitis specialists identified electronically using the AUS and Proctor Foundation e-mail LISTSERVs. Sixty-eight percent of respondents practiced in a university or academic setting and 73% had 6 or more years experience treating uveitis. One might presume, therefore, that the vast majority of the participants had both knowledge and experience regarding previously published uveitis treatment guidelines [11]. This view is supported by the fact that the median acceptable maintenance dose of oral prednisone reported by the respondents was 7.5 mg/day and was in all instances at or less than the 10 mg/day recommended by these same guidelines. A number of interesting observations were made by the authors. First, antimetabolites, most notably methotrexate and mycophenolate mofetil, tended to be the most preferred and first used immunosuppressive agents despite only modest effectiveness, both perceived [12] and actual—at least as measured by control of inflammation and corticosteroid-sparing success rates [13–15]. This approach appears to have come from complex considerations regarding cost, ease of administration, and availability of long-term efficacy and tolerability data. Conversely, the alkylating agent cyclophosphamide and the TNF-α inhibitors were both less preferred and less frequently used, despite higher perceived effectiveness. Concerns seemed to center around safety/tolerability in the case of cyclophosphamide, whereas cost, lack of long-term data, and to a lesser extent ease of administration were the major reasons for not prescribing TNF-α inhibitors. Respondents were not queried regarding the use of chlorambucil [16–18], etanercept [19–23] or the interferons [24, 25] despite reports of therapeutic benefit with these agents in selected patients with uveitis. Second, the most favored agent for the treatment of intermediate and posterior/panuveitis was mycophenolate mofetil, a preference supported at least in part by high success rates with this agent in the Systemic Immunosuppressive Therapy for Eye (SITE) Diseases Cohort Study [14, 15, 26]. Third, azathioprine was generally the least favored antimetabolite regardless of anatomical localization of the inflammation, despite the fact that a number of randomized trials comparing azathioprine to methotrexate in various rheumatologic diseases and to prevent organ rejection post-transplantation have shown similar efficacy and tolerability [12]. And fourth, cyclosporine, the only leukocyte signaling inhibitor queried, was rarely preferred and infrequently used, due primarily to concerns over tolerability. Such perceptions appear to be supported, at least partially, by the findings of the SITE Study group, which reported relatively low corticosteroid sparing success with cyclosporine [15, 27]. Moreover, adverse events tend to increase with age in patients treated with cyclosporine and are particularly common in the elderly [27].

Taken together, published practice surveys in uveitis highlight several important points. First is the need for improved education and wider dissemination of evidence-based reviews regarding uveitis treatment—particularly to non-specialists who care for a sizable proportion of patients with uveitis [28]. Second is the importance of practical and timely treatment guidelines promulgated by those most knowledgeable in the field—either as expert panel recommendations [11] or true consensus guidelines [29]. Perhaps surprisingly, such treatment recommendations or guidelines do not yet exist for ocular toxoplasmosis—the most common cause of infectious uveitis worldwide [30, 31], and while guidelines do exist regarding the use of immunosuppressive drugs in patients with noninfectious uveitis, they are largely unknown by all but those already quite experienced in the use of such agents. Moreover, current treatment guidelines for uveitis are more than 10 years old, provide little information regarding treatment goals or endpoints when using individual or combined therapies to treat specific conditions or complications of uveitis, and include relatively little mention of the newer biologic agents. It is perhaps time, therefore, to consider introducing evidence-based treatment recommendations or guidelines for ocular toxoplasmosis and updating existing guidelines on the use of immunomodulatory agents to treat ocular inflammatory disease, including recent data on the use of TNF-α inhibitors and the interferons. And third is the importance of standardized outcome metrics in uveitis treatment studies [15]. Perceptions and preferences are noteworthy, but are by no means substitutes for data obtained from well-designed studies, be they retrospective and observational or, when feasible and appropriate, prospective and randomized.
